# Microbiota Composition in Upper Respiratory Tracts of Healthy Children in Shenzhen, China, Differed with Respiratory Sites and Ages

**DOI:** 10.1155/2018/6515670

**Published:** 2018-06-14

**Authors:** Heping Wang, Wenkui Dai, Xin Feng, Qian Zhou, Hongmei Wang, Yonghong Yang, Shuaicheng Li, Yuejie Zheng

**Affiliations:** ^1^Department of Respiratory Diseases, Shenzhen Children's Hospital, Shenzhen 518026, China; ^2^Department of Computer Science, City University of Hong Kong, Kowloon Tong 999077, Hong Kong; ^3^Department of Microbial Research, WeHealthGene, Shenzhen 518000, China; ^4^Department of Infectious Diseases, Shenzhen Children's Hospital, Shenzhen 518026, China

## Abstract

The upper respiratory tract (URT) is home to various microbial commensals, which function as competitors to pathogens and help train the immune system. However, few studies have reported the normal microbiota carriage in the URT of healthy Chinese children. In this study, we performed a 16S rDNA gene sequencing analysis of 83 anterior nares (ANs), 60 nasopharynx (NP), and 97 oropharynx (OP) samples from 98 healthy children in Shenzhen, China (≤12 years of age). The microbiota in ANs and NP is the same at different ages and typical species in these sites include* Moraxella*,* Staphylococcus*,* Corynebacterium*,* Streptococcus*, and* Dolosigranulum*. By contrast, the OP is primarily colonized by* Streptococcus*,* Prevotella*,* Neisseria*,* Veillonella*,* Rothia*,* Leptotrichia*, and* Haemophilus*.* Streptococcus *and* Rothia *keep low abundance in OP microbiota of children** ≤**1 year old, whereas* Prevotella*,* Neisseria*,* Haemophilus*, and* Leptotrichia* amass significantly in individuals** >**1 year old. This work furnishes an important reference for understanding microbial dysbiosis in the URT of Chinese paediatric patients.

## 1. Introduction

The upper respiratory tract (URT) functions as an interface between exterior environment, lung, and gastrointestinal tract [1]. Several reports have demonstrated that the normal microbiota of the URT confers colonization resistance against pathogen intrusion and performs immune education [2, 3]. The lipopolysaccharide (LPS) of* Prevotella* antagonized the LPS produced by* Haemophilus influenzae* and inhibited Toll-like receptor 4- (TLR4-) mediated mucosal inflammation [4]. Th17 immune responses were activated by commensal bacteria and active metabolic products that primed mucosal immunity against respiratory pathogen colonization [5]. Various immune responses can be also induced by specific clearance mechanism of different pathogens [6, 7]. The expression level of interleukin 8 (IL-8) in pulmonary epithelial cells increased after exposure to* Moraxella *[8]. The expression of IL-6 and interferon *γ* (IFN-*γ*) mounted highly to inhibit the virus-associated inflammation [9, 10]. These findings suggest a significant role of URT commensals in immune maturation and clearing pathogens.

The URT microbiota varies dramatically with niches and developmental stages [2, 11–13]. Previous study [14] found that the nasopharyngeal microbiota differentiated as early as 1 week of age and stabilized after 6 weeks, with a predominance of* Moraxella*,* Dolosigranulum*, and* Corynebacterium*. Dominant* Haemophilus*/*Streptococcus* in nasopharyngeal microbiota indicated high incidence of respiratory infections [14]. A one-year longitudinal study on newborn infants indicated that the nasal microbiota was primarily shaped by age, with increasing bacterial density and decreasing diversity [15].

By comparison with healthy European and American children, URT microbiota in healthy Chinese children remains little explored. In this study, we conducted 16S rDNA analysis of 240 URT samples from 98 healthy children in Shenzhen where an increasing number of young families are residing in. We aimed to profile microbiota structure at anterior nares (ANs), nasopharynx (NP), and oropharynx (OP) as well as to conduct comparison among samples with different ages.

## 2. Material and Methods

### 2.1. Subjects Selection

Children were recruited to an examination room in Shenzhen Children's Hospital and all the volunteers' families were indigenous residents in different regions of Shenzhen. The inclusion criteria requested no asthma and family history of allergy, no history of pneumonia, no cough, fever or other respiratory/allergic symptoms one month before sampling, no respiratory infection and antibiotic exposure for at least 1 month prior to the study, and no respiratory symptoms 1 week after sampling.

### 2.2. Sample Preparation and Sequencing

NP, OP, and ANs microbial samples were collected by an experienced clinician with specific swabs (25-800-A-50, Puritan, Guilford, USA; 155C, COPAN, Murrieta, USA). The swabs, which were unused or opened in the sampling room for several seconds, were served as negative controls to evaluate potential contamination. All samples were stored at -80°C within 20 minutes after sampling.

DNA extraction was performed by PowerSoil® DNA Isolation Kit (MO BIO Laboratories). The DNA library of 16S rDNA V3-V4 region was constructed by the PCR amplification and sequenced on Illumina MiSeq Sequencing platform. All sequencing data were deposited in GenBank database under accession number SRP090593.

### 2.3. Bioinformatics Analysis

Raw sequencing data were processed through QIIME pipeline [16]. Data filtration, operational taxonomic units (OTUs) clustering, taxonomic classification, and diversity calculation were conducted following our previous study [17]. The same number of tags was utilized to construct rarefaction curve and assess the sequencing saturation of each sample. The confounding effects of various characteristics on bacterial composition were evaluated by the PERMANOVA [18]. URTs microbial samples were clustered following previous studies [11, 19, 20]. Bray-Curtis dissimilarity was employed to assess the similarity between microbial samples. Microbiota comparison between two URT sites was conducted through Wilcoxon rank-sum test and adjusted by false discovery rate (FDR) (*q*-value). All graphs were prepared by R (v3.2.3) (packages ‘ggplot2' and ‘NMF') and SVG (v1.1).

## 3. Results

### 3.1. Sample Characteristics, Data Output, and Confounder Analysis

In this study, we totally enrolled 115 children aged ≤12 years old in Shenzhen Children's Hospital through health examination. Ninety-eight children (50 girls and 48 boys) were selected after health examination and at least one-week follow-up (Table 1, Supplementary Table 1).

The concentration of extracted DNA in the unused sampling swabs and DNA extraction kits was lower than 0.01 ng/*μ*l, whereas it was higher than 80 ng/*μ*l in sampling swabs. In addition, 16S rRNA gene amplification on the extracted DNA exhibited less than 0.01 nmol/l bacterial DNA in the enveloped sampling or extraction materials, indicating negligible DNA contamination from sampling and DNA extraction materials.

High-quality tags produced from the ANs, NP, and OP microbial samples averaged 42,195 (17,102-68,499), 42,698 (19,923-88,084), and 32,331 (17,112-56,071) (Supplementary Figure 1), and the number of OTUs at ANs, NP, and OP averaged 250, 235, and 102, respectively. Confounder analysis indicated that age is the most significant factor to explain variations in microbial samples at each site (*p*-values for ANs, NP, and OP are 0.003, 0.013, and 0.001, respectively) (Supplementary Table 2).

### 3.2. ANs and NP Tend to Harbour a Similar Microbiota with Different Ages and the OP Differs from Them

**Figure 1:**
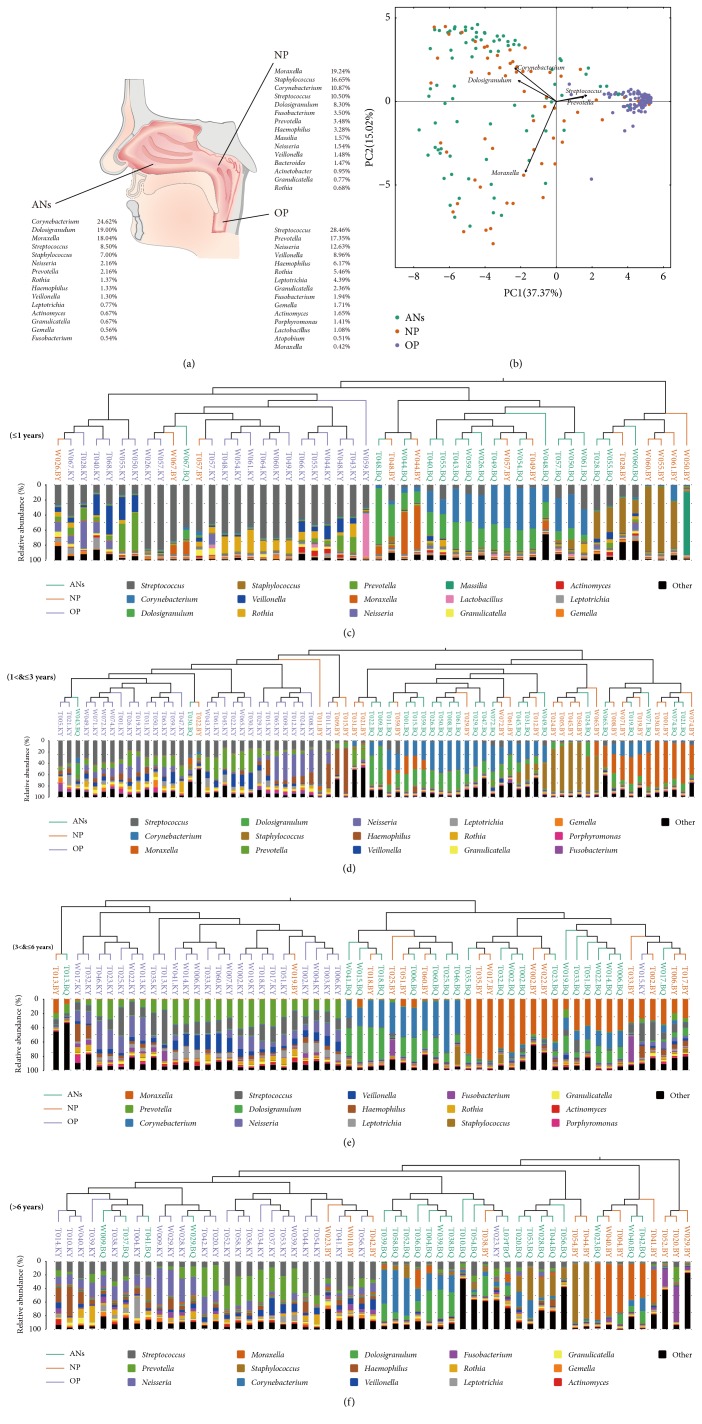
**Microbial flora in the ANs, NP, and OP are differentiated at different ages. (a) **The top 5 genera among the ANs and NP were* Moraxella*,* Staphylococcus*,* Corynebacterium*,* Streptococcus*, and* Dolosigranulum*, and OP harboured different dominant genera.** (b)** The microbiota composition of the OP remained different from those of the ANs and NP. In each subgroup (**(c)** ≤1 year old;** (d)** >1 and ≤3 years old;** (e)** >3 and ≤6 years old;** (f)** >6 years old), all OP samples exhibited an independent cluster other than mixed ANs and NP. Each genus was painted with specific colours.

*Corynebacterium*,* Streptococcus*,* Staphylococcus*,* Moraxella*, and* Dolosigranulum* dominate the ANs and NP microbiota while the dominant bacterial components at OP differ (Figure 1(a)). PCA indicates that ANs and NP microbial samples cluster differently compared to OP microbial samples (Figure 1(b)).

To further understand whether the ANs and NP microbiota were differentiated at specific age, microbial samples were stratified to four subgroups: ≤1 year old (21 children), >1 and ≤3 years old (28 children), >3 and ≤6 years (25 children) old, and >6 years old (24 children) (Figures 1(c)–1(f)). The dissimilarity between ANs and NP microbial samples is lower than that between ANs and OP microbial samples in each subgroup (Supplementary Figure 2). Moreover, OP microbial samples in each subgroup tend to be found in one cluster while ANs and NP microbial samples are clustered closely (Figures 1(c)–1(f)).

### 3.3. Predominant Microbial Phyla and Genera in the OP Differ Significantly from That in ANs and NP

Firmicutes are the dominant phyla in the ANs, NP, and OP microbiota (Supplementary Table 3). Actinobacteria account for 7.9%, 27.1%, and 12.5% of the OP, ANs, and NP microbiota, respectively (Supplementary Table 3). Bacteroidetes in the OP microbiota are 4.94-/2.68-fold higher than that in the ANs/NP (*q*-values <0.001) (Supplementary Table 3).

*Moraxella*,* Staphylococcus*,* Corynebacterium*,* Streptococcus*, and* Dolosigranulum *totally represent 65.6–77.2% of the ANs or NP microbiota (Figure 1(a), Supplementary Table 4). The predominant bacterial components at OP are* Streptococcus*, followed by* Prevotella*,* Neisseria*,* Veillonella*, and* Haemophilus*, the sum of which only account for <10% in the ANs/NP microbiota (Figure 1(a)).

### 3.4. OP Microbiota Develops Dramatically during First Year and Then Turns to Be Stable

**Figure 2:**
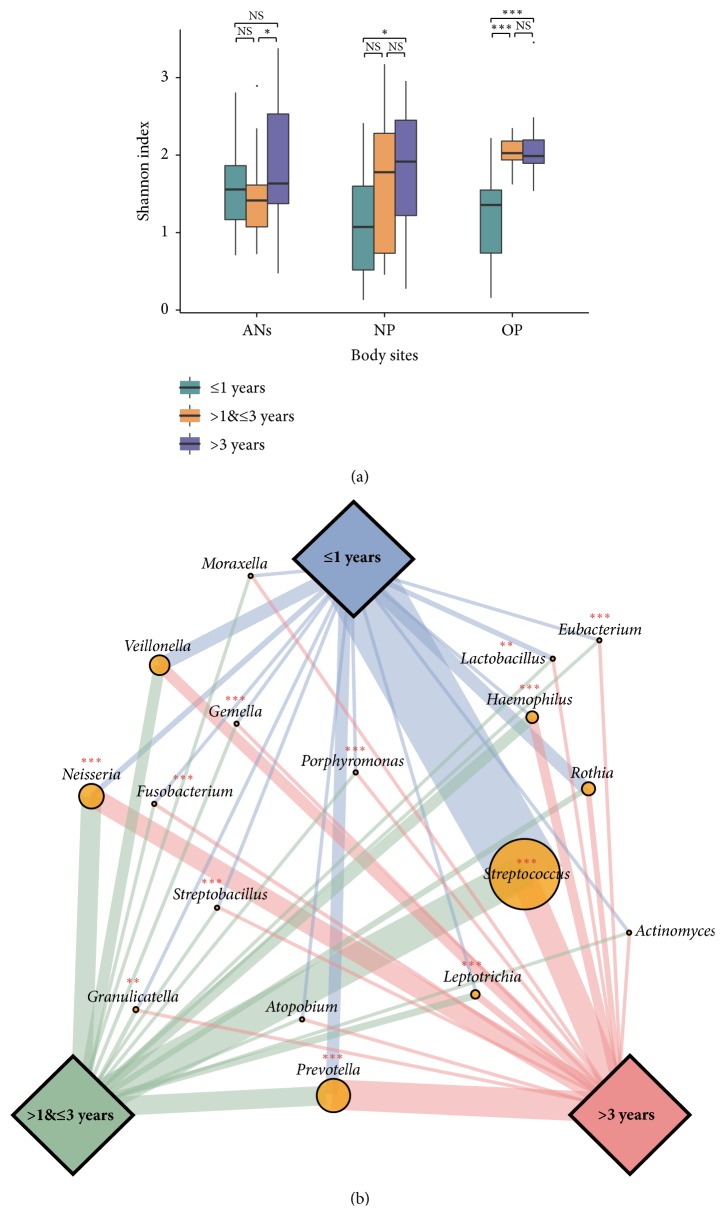
**Microbial diversity in the ANs, NP, and OP at different ages and dominated genera in the OP of children ≤1, >1 and ≤3, or >3 years old. (a)** The microbial diversity in the OP increased significantly during the first year of life and then remained stable, ANs and NP shown to be similar microbial diversity at different age. NS, *∗*, and *∗∗∗* represent* p*-value >0.05, ≤0.05, and ≤0.001, respectively.** (b)** Structure of dominant genera based on relative abundance. Each circle represents genus with total relative abundance in the three groups, and the width of line represents genus with the relative abundance in each group. *∗∗* and *∗∗∗* represent* q*-value of Kruskal-Wallis test ⩽ 0.01 and ⩽ 0.001, respectively.

The diversity of the OP microbiota increased dramatically in the first year (*p*-value <0.001) and kept stable after one year old (Figure 2(a)). By contrast, the bacterial diversity in ANs and NP microbiota revealed less change than that in OP microbiota (Figure 2(a)).

We then stratified the OP microbial samples to three subgroups to understand how the predominant genera distributed in children with different ages (Figure 2(b)). Except for* Veillonella, Rothia, Actinomyces, Atopobium, and Moraxella*, the dominated bacterial components in OP of children ≤1 year old differ significantly from that in other two subgroups (Figure 2(b)). None of the top fifteen bacterial genera in OP microbiota alters overtly after 1 year old (Supplementary Table 5). Genus* Streptococcus* is the most abundant in children ≤1 year old (52.7%) but diminishes to 25.7% (>1 and ≤3 years old) (*q*-value < 0.001) and 19.5% (>3 years old) (*q*-value <0.001) (Supplementary Table 5), accompanying with* Veillonella, Rothia,* and* Lactobacillus* declined with age (Figure 2(b)) as well.* Neisseria*,* Haemophilus*,* Leptotrichia,* and* Prevotella* are four OP predominant genera, which increase from 0.6~9.3% to 4.2~22.6% in children >1 year old (*p*-values ≤ 0.05) (Supplementary Table 5).

## 4. Discussion

The URT could filter and humidify inhaled air and is colonized by various microbes [21]. ANs and NP microbial commensals primarily extract nutrients from the respiratory epithelium [22, 23] and are easily affected by the skin and external atmosphere. These findings suggest a low diversity in the ANs/NP microbiota and highly enriched* Corynebacterium* and* Staphylococcus*, which are dominant in the skin microbiota [3, 24].* Moraxella*,* Dolosigranulum,* and* Streptococcus *are three typical genera in the ANs and NP, which is in accordance with previous reports [2, 11–14]. Microbial exposure aids in the development of host immune tolerance in early life [25, 26]. ANs/NP microbiota-accumulated* Corynebacterium* and* Dolosigranulum *were associated with lower risk of acute otitis media and respiratory infection [27], partly explaining the predominance of* Corynebacterium* and* Dolosigranulum* in ANs and NP microbiota of selected children. Given the frequent exchange between ANs/NP and exterior environments, various reports demonstrated rapid assemblage of ANs and NP microbiota in the first month of life [14, 15, 28]. In addition, delivery mode and feeding type only imposed effect on NP microbiota in 6 months after birth [14]. Considering small number of children ≤6 months old, our study found little discrepancy of ANs/NP microbiota among children with different ages.

In contrast with the ANs/NP, food ingestion and oesophageal reflux may affect the OP microbial composition, implicating a distinct and more complex microbiota structure in the OP [13, 29]. Our study suggested the development the OP microbiota in the first year of life, which is a so-called critical window for airway development and immune maturation [19] as well as gastrointestinal tract [30]. Previous study identified similar predominant genera in the OP of Canadian children aged 1 to 4.5 years old [31] and OP microenvironment gradually changed with respiratory epithelium development and diet alteration [1, 32], which may trigger later maturation of the OP microbiota compared to ANs and NP. Correspondingly, the microbiota diversity and predominant microbial colonizers of the OP keep stable after 1 year old, which resembles gut microbiota assemblage [32].

URT microbial commensals participate in a stable interaction network under healthy conditions [21] and impaired URT microbiota seemed to predispose to pathogenic infections [13, 21, 29]. Prior study demonstrated that* Streptococcus-*dominant NP microbiota was extraordinarily associated with allergy [11].* Haemophilus*-dominant NP microbiota indicated the high severity of bronchiolitis [33] and the NP microbiota impacts the severity of lower respiratory infection (LRI) [28].

Findings in this study will provide a healthy reference of URT microbiota in a typical southern city in China and boost the understanding of airway diseases and underlying microbial etiology. However, there also exist several limitations, including the shortage of newborn infants, single-centre sampling, and the small sample size of the cohort. Otherwise, a large cohort and longitudinal study of the URT microbiota is also considered in our laboratory.

## 5. Conclusions

This study provides a critical reference for the normal URT microbiota as well as the site- and age-specific URT microbial structure of healthy children in Shenzhen, China. Additionally, this work will facilitate the understanding of the microbial aetiology in respiratory diseases of Chinese paediatric patients.

## Figures and Tables

**Table 1:** Sample information.

	**Healthy Children ** **(n=98)**
**Gender**	
** Female**	50
** Male**	48
**Age(year)**	3.1(0.1~10.8)
**Height(cm)**	97.5(50.0~140.6)
**Weight(kg)**	15.25(3.45~38.2)
**Delivery mode**	
** Cesarean section**	31
** Vaginally born**	67
**Feed pattern**	
** Breast feed**	31
** Breast + Milk feed**	54
** Milk feed**	13
**Living environment**	
** Urban**	43
** Suburb**	37
** Rural**	18
**Family history of allergy**	0
**History of pneumonia**	0
**Asthma**	0
**Cough**	0
**Fever**	0
**Wheezing**	0

## Supplementary Materials

Supplementary 1
** Supplementary Tables. Table S1**: sample information;** Table S2**: confounder analysis;** Table S3**: intergroup comparison at phylum level: NP-OP, ANs-OP, and ANs-NP;** Table S4**: intergroup comparison of dominant genera: NP-OP, ANs-OP, and ANs-NP;** Table S5**: comparison of dominant genera in OP among different ages.

Supplementary 2
** Supplementary Figure 1:** rarefaction curve of the OTU number to the tag number. All samples' rarefaction curve reached plateau, which demonstrates that the data is enough for further analysis. The X-coordinate represents the selected tags, and the Y-coordinate stands for the relative OTU number.

Supplementary 3
** Supplementary Figure 2:** dissimilarity of bacterial community in ANs-NP and ANs-OP. The X-coordinate represents the 4 subgroups with different ages, and Y-coordinate stands for the dissimilarity of bacterial community. *∗∗∗* represents* p*-value ⩽ 0.001.
